# Psychological Consequences in Patients With Amputation of a Limb. An Interpretative-Phenomenological Analysis

**DOI:** 10.3389/fpsyg.2021.537493

**Published:** 2021-05-26

**Authors:** Andra Cătălina Roșca, Cosmin Constantin Baciu, Vlad Burtăverde, Alexandru Mateizer

**Affiliations:** ^1^Department of Orthopedics and Traumatology, National University of Political Sciences and Public Administration, Bucharest, Romania; ^2^Department of Sociology-Psychology, Carol Davila University of Medicine and Pharmacy, Bucharest, Romania; ^3^Faculty of Psychology and Educational Sciences, University of Bucharest, Bucharest, Romania

**Keywords:** chronic condition, limb amputation, psychological consequences, interpretative phenomenological analyses, negative affectivity, self

## Abstract

The study aimed to identify the psychological changes that result from the amputation of a limb and the ways in which patients coordinate their daily lives. The study uses an interpretative phenomenological analysis (IPA) aimed at understanding individual experiences in seven patients who have suffered limb amputation. The method used consisted of individual, semi-structured interviews, conducted approximately 4 months after surgery, to patients at home or in hospital, at the time of their regular checkup. The interviews were audio recorded, transcribed and, following the qualitative analysis performed, six common themes were identified: emotional impact, negative affects, tendency toward isolation, role constraints and limitations, phantom limb, and emotional balancing. A specific theme for patients who have suffered amputations is phantom limb pain, which has received special attention from researchers. The last topic relates to the tendency toward emotional balancing and psychological calibration to return to normal life.

## Introduction

Amputation is traumatic both as a surgery itself and also due to its consequences. Perceived as an aggression to bodily integrity, besides physical suffering, it can initiate or aggravate a series of disharmonies that disrupt the patient’s well-being. Desmond and MacLachlan (2002) consider that amputations cause considerable changes in everyday life of the patient, and especially in psychosocial relationships. Physical disability can lead to despair, depression, nervousness, anxiety, loss of self-esteem, stigma, isolation, and the recognition of weakness (Khan et al., 2018). The decision to amputate a limb is difficult for both medical staff, the patient, and his/her family (Boccolini, 1995). The fear of losing a visible part of the body leads to a true sense of body disintegration (Bergo and Prebianchi, 2018). However, amputation may be necessary to save a life. That is why a closer approach to this issue is needed.

Amputation may have multiple causes including burns, frostbite, peripheral vascular disease trauma, infections, and tumors (Simsek et al., 2017). As a surgical procedure, it is done to save life, control pain, malignancy or gangrene in an affected member (Waqar et al., 2015). It can also have a preventative role. An 18 months longitudinal study, according to the WHO (Seidel et al., 2006), found that 14.1% of the amputees reported depression (5.8% in males and 9.5% in females). Another study (McKechnie and John, 2014) found that the levels of anxiety and depression are significantly higher in amputees than the general population. Horgan and MacLachlan (2004) estimate that depression and anxiety are moderately elevated up to 2 years after amputation. Physicians should take this into account in addition to amputation treatment.

This study confirms the meta-analysis of Bergo and Prebianchi (2018) who, quoting Rodrigues (2011), concluded that the emotional responses following amputation differ from patient to patient, and are influenced by a constellation of factors: the etiology of amputation, elements of personal life, the social-historical moment, the pathogenicity of the disease, and the patient’s interpretation of the symptoms. Patients who suffer from amputation due to a vascular disorder that causes unbearable pain will feel relieved. Similarly do those who, in this way, hope to attain the healing of bone cancer. Conversely, those traumatized react differently and live the uncertainty of their future prospects. The sooner a prosthesis is applied, the associated psychological problems diminish.

Knowing the psychological consequences of limb amputation is useful for practitioners in the medical field because it helps them develop effective counseling and rehabilitation programs. Various studies investigated the psychological consequences of limb amputations (Khan et al., 2018). Almost all of them relied on a cross-sectional design by administering psychometric questionnaires to the participants. Even if this method generates important empirical evidence when it comes to the psychological consequences in patients with amputation of a limb, it has its limitations. For example, such an approach, limits participants to the information captured by the psychometric measure, while information that may be relevant to psychological consequences of limb amputation, not encompassed in the measure, cannot be reported by participants. Therefore, we think that pursuing a qualitative approach, such as the interpretative phenomenological analysis (IPA) would provide richer and more insightful information, which should help to better understand the consequences of limb amputation.

Even though the previous studies on this topic offer valuable information in understanding the various psychological consequences of limb amputation, these studies did not provide a robust theoretical framework that could serve for the interpretation of the identified psychological consequences. We consider that Self psychology model of Kohut (1966) may be used as a theoretical framework to explain the psychological consequences of limb amputation. This theory describes the self as a collection of self-representations. These collections are understood in terms of representing the cognitive-affective structure of one’s identity, being named “the representational world” – that is, people’s representations of the self and others (Sandler and Rosenblatt, 1962). This representational world develops as a consequence of the interaction between the individual and his parents, family and the people around him in his childhood (Kohut, 1966). This theoretical model describes the self as bipolar, with ambitions on one side, ideals on the other, and talents and skills driven by these two poles arched between them. The degree to which individuals develop these characteristics depends on the extent to which their caregivers, in the first years of life, are empathic in their response to the children’s need for mirroring, support and idealization of caregivers, thus transmitting their own sense of security and self-esteem to them (Kohut and Wolf, 1978).

Although the self of one individual is stab across adulthood (Markus and Kunda, 1986), it may be subjected to important changes and variations as a result of traumatic life experiences (Saakvitne et al., 1998). Because limb amputation is perceived as traumatic (Desmond and MacLachlan, 2002), we consider that it should lead to shifts and modifications on the individual self-structure. Because it affects the integrity of the body, the talents, and skills, which are components of the self-structure, are affected. As a consequence, the “representational world” of the individual is subjected to important modifications. Therefore, the affected individual perceived himself as different and usually inferior compared to the period before amputation, and this perception may activate negative cognitions associated with catastrophizing regarding his future functionality and adaptation. These negative cognitions usually lead to exaggerated negative affectivity such as anger, anxiety, hostility, or depressive tendencies (Beck et al., 2005). Further, negative affectivity tends to lead to maladaptive behaviors (Beck, 2011).

Therefore, relying on the previous rationale, we hypothesize that the psychological consequences of limb amputation can be clustered in negative cognitions such as catastrophizing, negative affectivity such as anger, anxiety, hostility, or depressive tendencies, and maladaptive behaviors.

## Materials and Methods

### Method

The IPA aims to understand each individual experience regarding certain events or common processes. Smith (1995) identifies two aspects of this qualitative method: phenomenology and symbolic interactions. Far from being spectators of their own lives, Smith (2007) believes that individuals are self-analyzing by wanting to understand what is happening to them, by personally and subjectively interpreting the events they are involved in. An IPA study implies an intensive interpretation of the information obtained from a small group of participants linked to a common event in their lives. Semi-structured interviews, focus groups, or journals provide data, which can reveal a comprehensive psychological portrait with generalization value when scientifically processed.

The study was conducted according to the guidelines of the Declaration of Helsinki, and approved by the Institutional Ethics Committee of NUPSPA (protocol code no.145, 01.04.2019).

### Participants

Specific to qualitative research is the low number of participants, in order to be able to carry out an in-depth analysis of individual experiences. Smith et al. (2009) believe that a number of 3–6 participants in an IPA study would be sufficient. In this case, a number of seven participants, patients of a traumatology hospital in Bucharest, aged between 41 and 75 years, were selected. They were offered the opportunity to volunteer for a research concerning the experience of amputation. In order to ensure confidentiality, an identification number was randomly assigned to each participant and age was considered only for the purpose of establishing a range. All respondents were made aware of these procedures and gave verbal consent to participation. Within this group one participant had the left upper limb amputated, while the rest of the participants had one of the inferior limbs amputated. The causes for amputation were various and are shown in Table 1. At the time of the interview none of the participants had yet been given a prosthesis.

**Table 1:** Participants’ description.

Participant	Amputation cause
1	Left-side calcal necrosis. Obliteratory arteriopathy of the inferior left limb
2	Myosarcoma inferior left limb
3	Inferior right limb toe necrosis. Unbalanced type 2 diabetes mellitus
4	Traumatic amputation upper left limb
5	Ischemic gangrene inferior right limb
6	Traumatic below knee amputation of inferior left limb
7	Amputation of inferior left limb

### Procedure

#### Data Collection

After giving consent, the participants were interviewed (audio recorded) for about an hour at the hospital or at home. Data was collected in the form of a semi-structured interview, with a flexible interview schedule. The interview schedule was developed according to IPA methodology (Smith et al., 1999, 2009) in order to facilitate a comfortable and productive interaction with the participants. Starting with one research question about the “*emotional experience of the individual after amputation of a limb*,” five main topics were selected to provide the overall structure of the schedule. After a consensus was reached between all researchers, the interview was finalized. Its function was to guide and not to prescribe the topics covered by the interview. For each question, prompts have been prepared in case a participant has difficulties responding, and to offer a range of possible directions for investigation. Following IPA methodology and our prior considerations the interview guide was not referred to directly when conducting the interviews. The interviewer’s knowledge of the interview guide enabled enough flexibility as to permit substantial room for expression on behalf of the participants within the proposed framework of all investigation. All of the interviews were conducted by one of the authors (CR) who was not known to any of the participants prior to the research interviews. No further inquiries were needed in order to achieve data saturation. All interviews were transcribed verbatim by one researcher (AM).

### Data Analysis

Using IPA (Smith et al., 1999, 2009; Smith and Dunworh, 2003; Reid et al., 2005; Smith, 2007), the extraction of emerging themes was done repeatedly until raw, unprocessed themes were obtained (Smith et al., 2009). The transcripts were read line-by-line, by each individual researcher, focusing on the research question. After completion, the interpretative notes were analyzed to extract themes and sub-themes of interest in relation with the research question. Quotations that were relevant to the extracted themes were then selected and, in the final step, the data was reviewed against all transcripts. This procedure allowed the extraction of recurrent themes about the understanding and meaning that participants give to the amputation experience.

## Results

Following the analysis six themes have been highlighted: (1) emotional impact, (2) negative affects, (3) tendency toward isolation, (4) role constraints and limitations, (5) phantom limb phenomenon, and (6) emotional balancing. The examples used in this section were considered to be representative for the themes in question or for a particular manifestation related to these themes.

### Emotional Impact

Regardless the reasons for amputation, whether due to traumatic causes or as a consequence of illness, the emotional shock exists. It may have a smaller or larger amplitude depending on a variety of factors such as patient age, medical culture, medical cause etc. As a result of amputation, the research participants’ reports were loaded with drama. The first emotional response to amputation was one of despair, a severe sense of self-collapse, something almost unbearable. In this context, the wish for death, as an expression of extreme dread, was mentioned by many of the participants.

“*I’m a lost man! It will not be how it was!*” (Participant 3)

“*… awakening from the (induced) coma was a nightmare … I could not move … I wanted to get away … it was terrible! I felt I was in a prison and I could not run. I could see my right hand, but I missed my left hand. Why? For a moment I wanted to die. God, take me! Lord, I cannot live like this!*” (Participant 4).

“*Well, what can I say … I thought the sky will fall on me … I started crying, I did not want to talk to anybody*” (Participant 2).

“*At first, I said no, I refused … I thought I’d rather die than… I even told the doctor … I’d rather die than lose my leg*” (Participant 1).

### Negative Affects

#### Anxiety

After surgery, for an extended period of time, the interviewed patients noticed the appearance and increase of anxiety. A lot of negative thoughts invaded their minds. Projections about the future were grim, marked by sadness, helplessness, and even despair. Existential uncertainty, lack of control and further anticipated losses in one’s life due to amputation were the primary causes of anxiety and consequently ruminations and insomnia.

“*We had a time when we were having sleepless nights. I’m still anxious. I’m thinking what’s going to happen to me if my wife dies before me. Besides her, I have no one else. Now she lifts me in her arms, puts me in the wheelchair, but if I remain alone, what will I do?*” (Participant 5).

“*I have insomnia … I can only sleep with medication … I’m anxious. I do not know what’s going to happen to me in the future. I have a terrible state of insecurity*” (Participant 4).

“*I could not sleep more than two hours a night. I’m taking sedatives. I did not accept the idea that I do not have two legs anymore. I was very worried. I was afraid I’d be kicked out of my job. And now, when I have to start work, I wonder if I will be able to handle it*” (Participant 6).

#### Anger and Hostility

The amputation of a limb is associated not only with physical loss and change in body image but also with an abrupt severing in one’s sense of continuity. For participants with amputation as a result of physical trauma the event is often experienced as a transgression and can lead to frustration and anger.

“*I get angry when I think I can’t do what I was doing before*” (Participant 6).

“*… a terrible frustration. Things were taken from me, my job, my independence … it’s a terrible frustration but I am fighting it*” (Participant 4).

“*… I sometimes get angry because I can’t do what I did before … knowing that you could do something without asking for someone’s help, now I can’t even … it’s, yes, a sort of … I get angry when I realize that now I need to call someone for things I used to be able to do on my own before … but I will get used to it, what else can I do?*” (Participant 1).

Hostility toward others makes sense in the context of amputation. Frustration at the loss of a limb can generate a feeling of bitter indignation at having somehow been treated unfairly by fate. Thus, others are at risk of becoming targets of resentment and envy of their “wholeness.”

“*a disabled person becomes more selfish, mournful, mischievous because she is distressed in her own way*” (Participant 5).

### Tendency Toward Isolation

#### Struggle With Depression

Loss of the sense of control left the participants feeling exposed and vulnerable, emotionally and physically, to a world that now seems more dangerous than ever. This abrupt change in one’s self state is experienced as a regression to a dependency phase that has crippling emotional effects in the absence of sufficient internal and external resources. Overwhelming distress causes depression and severely hinders the recovery process. Participants with an effective adaptive capacity seemed to fight against depression and to recognize it’s damaging capacity.

“*I cannot afford to fall prey to despair. I’m alone and I have to raise a child on my own. I have to fight. My little girl saw me cry twice, when I could not cope anymore*” (Participant 2).

“*I have a tortured life. I’m trying not to fall into depression. Looks like I need psychological counseling. I am a strong person on the inside. That does not mean it did not affect me and I do not have moments of despair. But with whom I have to fight … to fight continuously*” (Participant 4).

In the case of elderly participants, a sense of surrender in the face of adversity was present. Given that the feelings of dependency, lack of control and vulnerability are accentuated with age, elderly people already find themselves in a process of adjustment, having to reconcile old and new self-representations. The trauma of amputation in such circumstances can be defeating.

“*I pray to God I leave this world quicker … so I don’t torture my wife anymore*” (Participant 7).

“*If I was to die, I wouldn’t have any regrets … I’m 75 years old … it’s enough*” (Participant 5).

#### Guilt

For some participants amputation of a member was accompanied by the development of a guilt complex. Considering individual psychodynamics, guilt can be a cause and a powerful fuel for depression. In our findings, guilt was related to the realization of a link between their choices and the loss of a limb.

“*It was only my fault: alcohol. Diabetes brought me here*” (Participant 3);

“*I smoked for 40 years. That’s why*” (Participant 1).

“*I am guilty. I got off the tractor and did not stop the milling cutter. It was a big mistake. You are not allowed to repair a piece of equipment while functioning*” (Participant 6).

While for one participant, guilt seemed to be needed in an attempt at making sense of the trauma (car accident), in such a way that crucial resources for self-cohesion (in this case religion) remain intact.

“*I am a religious person. I shouldn’t have gone on a trip during Easter … I should have had a different state of mind … not going on a trip*” (Participant 4).

#### Social Withdrawal

In the case of some participants, the unpleasant image they present to others, the pity that they read in the eyes of friends and acquaintances, further their depression and leads them to social avoidance and retreat. The loss of a limb is also experienced as a narcissistic wound, a dent in the participants’ representation of themselves as autonomous, intentional, capable, desirable, and participative beings. This makes them prone to shame.

“*The fact that I do not have an arm made me isolate myself … I’m embarrassed by the situation I’m in. I do not want them to feel pity for me*” (Participant 4).

“*After surgery, I shut down my phone because it rang very often and when it did, I started crying badly when I was seeing who was calling me. What should I say to them? That I do not have a leg anymore? And what would they say to me? … That’s why I’d rather not answer them. Now when a friend comes and asks … I’m not going into details … exactly what happened etc. … I’m trying to avoid the subject. It hurts me*” (Participant 6).

“*I’m talking to my brothers on the phone. But they are busy. I do not want to get in touch with a lot of people so they’ll say „look at this poor fellow!*” (Participant 1).

At the same time, the need for convalescent isolation comes in conflict with other important necessities, such as resuming previous roles.

“*A job I was doing with pleasure (teacher) was taken away from me. How can I appear before my students without an arm?*” (Participant 4).

“*I was selling my vegetables at the market. The first time I went with the crutches it was unbearably shameful… People knew me … But I went. What could I do? I had to go ahead and do it*” (Participant 7).

For another participant, the personal feeling of helplessness and futility was projected outward. While it might not lead to physical isolation, it can, however, create serious barriers in relationships and engaging external resources.

“*I’m still going out and talking to a neighbor on a bench. I do not tell them anything about me … how can he help me? … But winter is coming, what will I do?*” (Participant 7).

### Role Constraints and Limitations

#### Struggle With a New Normal

Perhaps the most painful thing is that, before a prosthesis, the daily life of the patient with an amputated limb changes dramatically. Certain routine activities can no longer be carried out and with this comes a loss in roles and their contribution to one’s self representation.

“*I suffer that I can’t dress properly…*” (Participant 4)

“*I was taking care of my grandchild who was a year and eight months. He is really restless. I’d go out with him in the park. Now how can I run after him? I had to give up*” (Participant 3).

In addition to having a hard time doing domestic and professional activities, some participants claim to have dropped hobbies and previous physical activities. Also new constraints in one’s life now have to be acknowledged and dealt with. For one patient, the struggle to accept the new reality of his options is evident.

“*I was lively … I’m not the man to stand in one place. I always had to do something. I went on trips to the mountains and the sea. Now I cannot. How do I climb up the mountain or how can I go to the beach without a leg?*” (Participant 1).

#### Resuming Previous Roles

Focusing on and capacity to reengage in previous roles seemed to provide, for some participants, a shielding effect from the adverse impact of the trauma. If they are active and animated by positive thinking, they can better overcome negative affects and find the opportunity to recover a sense of utility and capacity.

“*Until now I was helping others. My mother was paralyzed in bed for years and lived with me. I took care of her. She went last year. Now her sister lives with me and she is almost blind. I’m helping her … just like that … without an arm*” (Participant 4).

Fear of losing the active role part of mother seems to be a driving factor in the case of one participant. In order to avoid traumatizing her daughter, she feels compelled to hide her emotional experience from her and continue with previous activities.

“*My daughter… she saw me cry maybe once or twice. She said ‘Please, don’t cry! Who’s the strongest and bravest on this planet?’ and I tell you, this got me… And friends who came and helped me and offered to spend time with my daughter but I wanted to continue our activities so that she does not feel that… She already has a father that she cannot count on, I don’t want her to feel the same about me*” (Participant 2).

The importance of maintaining a link with the former self (before trauma) through resuming previous roles is clearly expressed by one patient. He states that in doing so he will avoid losing his mind. Also, he manifests a desire to exert control over his trauma by engaging in the activity that caused his amputation.

“*I’m still using the tractor. But the wife and the children did not let me operate the milling cutter. It cut my leg off! They called my brother-in-law to operate the milling cutter. I was annoyed and said, ‘Leave me, do not try to convince me!’ … She pulls me back all the time. I’ll go crazy and it will be worse!*” (Participant 6).

#### Social and Family Ties

There is a difference in coping for participants assisted by family members and those who are alone. In the case of the former, we found the same feeling of embarrassment and frustration. Feeling helpless and unable to fulfill the role of provider, one participant had difficulties in accepting the support his family offered.

“*My whole family was next to me… my wife and father-in-law took turns staying with me at the hospital. I was embarrassed. Instead of me helping them, they were helping me*” (Participant 6).

With little to look forward to, an elderly participant was deeply affected by his state of dependency, which he saw as a hinder to his family.

“*I never thought I would become a burden for my family*” (Participant 5).

In the case of those who live alone or do not have a solid system of support in place, it is much more difficult. The feeling of being abandoned by friends and family might seriously affect their psychological recovery. Some participants expressed sadness at the idea of being abandoned and losing social support. Also, there was a reluctancy in asking for help.

“*At first, the few friends and neighbors took great care of me. Now they’re coming less and less often. They also have their problems*” (Participant 3).

“*I am a widower for 11 years. I do not have children. I have three brothers and two sisters, all married and with families. In the hospital all came to see me. There was no room in the hospital ward. Don’t worry, they would say, it’ll be good, we’ll help you. Then they got busy … I rarely get a phone call. I get the help of a niece. But she is busy as well … She has her concerns*” (Participant 1).

“*I had a lot of support … Friends were there for me during this really difficult time and also acquaintances and students of mine … there was always someone. It was a strong support for me that I was not left alone. Now a little less … they have their lives and they do what they can. But I need someone on a regular basis not on and off, you know?*” (Participant 4).

### Phantom Limb

#### False Sensations

Only two participants in this research, Participants 3 and 7 claim they have not had false perceptions about the amputated limb. The other participants either had these manifestations immediately after surgery or still have them. For one participant, the phantom limb phenomenon was the cause of new injury.

“*After I got home, I woke up one morning, I was rushing and when I got out of bed I fell straight onto the stump. They took me back to the hospital. Another misfortune … and now almost a year after surgery, I tend to sit one foot over another as I once did*” (Participant 6).

Other participants find these false perceptions to be a hindering factor in their daily activities. When implicit memory kicks in and habitual reactions take over one’s mode of being, they are again confronted with their new reality.

“*Yes, I feel it now. I’m tickling my toe, my thigh, my fingers … I want to massage it, but I realize it’s not there. If someone sits beside me on the bed, I tend to protect it. I am stressed because it physically doesn’t hurt, but it does psychologically, and I say: leave me alone, ghost!*” (Participant 1).

“*After the (car) crash I felt pain and my hand getting numb, I still have the numbness. I’ve been taking drugs for nine months now. I try to change my thoughts and then the pain stops, but the numbness remains. I tend to lean in it, grab the objects with both hands, cut my nails …*” (Participant 4).

One participant reports false sensations especially when he gets angry or at night.

“*I feel chills, pain …*” (Participant 5).

#### Body Representation in Dreams

Also, in relation to body representation, some participants reported dreams in which their bodies were intact. Their dreams seem to express a desire for erasing the trauma and regaining normality.

“*I dream that I have my left arm … that I am normal. I dream I am whole … I never dream of me without my arm*” (Participant 4).

“*In my dreams I have my leg, I’m running… I never dream of myself without my leg*” (Participant 2).

### Emotional Balancing

#### Determination

Getting accustomed to the new condition made some participants slowly come back to their previous mental state, and even hope that 1 day they will be almost as they were. One participant showed a strong drive toward recovery and overcoming the obstacles that his new condition brought. A strong will to overcome the trauma and repossess what he felt was taken from him.

“*Now, yes, I’m past this … I do not want to remember. I have to go ahead. I changed my car. I got a special one so I don’t have to rely on others. I want to take my life back*” (Participant 6).

For another participant, exposure to successfully adapted people in similar situations seemed to have a positive psychological effect. The idea of the prosthesis revived the possibility of a reduced gap between the old and new self. Also, in this case, other beneficial aspects were acknowledging the trauma and focusing on what remains rather than on what was lost.

“*Deep down I am optimistic. Even if I’m alone, I hope I can handle it. There are so many people living worse than me, with both legs amputated or an arm and a leg … I happened to meet a former colleague who had an accident 27 years ago. He got hit by a train and his left leg had to be amputated, just like mine. He has a prosthetic one and walks as if he was normal. He told me that some things change, but if I take it seriously, I will succeed …. We must believe in something. I know I’m not going to grow my leg back, but if I can get along well with the prosthesis, and … I’m not going to run, but do the things I need … I’ll be happy!*” (Participant 1).

#### Hope

Hope, determination to prevail, putting your faith in something, internal or external, and the capacity for humor seem to constitute strengths in the adjustment process of some participants.

“*God? … If He makes miracles He should have done so before my amputation … now all I can do is pray for my leg to grow back! (laughs) … and that’s not going to happen. But man must believe in something, if we would not believe in something then we could not hope … So, I live with hope, as any man… not to grow my leg back but to be able to accommodate to my prosthesis … not running, but, you know, to be able to do what I need, even if slower*” (Participant 1).

Feeling acknowledged and valued by her social environment, one participant regained hope for the future. Insofar as events in one’s life are believed to be a manifestation of God, this positive context allowed her, through her beliefs, to maintain self-cohesion and to gradually reengage in her former life.

“*I perceived it as a supernatural intervention … I mean, I did a lot of good in my life. God knows the good that I have done. And God did not forsake me, that’s clear … because when I went back to my work from recovery everyone was there for me and that was extraordinary … Joy and gratefulness*” (Participant 4).

“*In fact, faith has helped me. I rely on God. If I did not have this belief, I think I would have gotten to despair*” (Participant 4).

## Discussion

The main purpose of this study was to explore psychological consequences in patients with limb amputation. Six main themes emerged from the qualitative data, each with related secondary themes. Given the relative short amount of time passed since amputation (approximately 4–8 months on average), the main focus of the participants was found to revolve around acceptance of loss, mourning, attempts at dealing with negative emotions and regaining their autonomy. The process of constructing a new self-representation was thus in its early stages with most participants still adjusting to the new reality of their life. In this stage, the patients struggled with their new physical appearance, functional limitations, uncertainty about the constraints on their future opportunities, activities, personal agency, social ties, and the impact that these changes have on their internal representational world. For some participants in our study, this impact seemed to be devastating thus leading to a state of denial and continual struggle with negative affects and cognitions. As such, they gave the impression of being stuck in the initial shock and unable to engage in a healthy mourning process and available resources. At the same time, other participants manifested traces of hope for the future and determination to regain a sense of agency and self-worth. One important factor to consider here is the absence of the prosthesis among the participants. According to findings of Lundberg et al. (2011), the experience of the prosthesis ranges from viewing it as a valuable tool to almost becoming a part of the body. It seems that, apart from being a functional element, the prosthesis can also facilitate a psychological continuity, or link with the former self-representation, perhaps easing the transition and integration of a new self-representation.

### Initial Impact

In general, amputations are performed due to traumatic causes or planned medical reasons (Ali et al., 2017), the most favorable situation being when the patient is convinced that only by amputation, he/she can survive and thus wishes to amputate, as was the case for two of the participants in this study. The unexpected sudden loss of a member without prior counseling severely disrupts the psychological harmony of the patient. The results in this study show similarities to findings in other studies regarding the emotional reaction to amputation. Patients can have different responses such as sadness, hate, shock, anger, suicidal ideation, or non-acceptance of the situation (Senra et al., 2012). In our study, participants reported experiencing a state of dread after surgery, one that seriously threatened the integrity of the self, almost as if the loss of a limb was announcing the danger of losing self-cohesiveness, a state of disintegration of the self. This in turn enables the mobilization of defenses against this intolerable state, looking to restore a sense of internal coherence and vitality. It is within this psychological setting that participants managed to view their loss as a partial one and gradually relate emotionally to it in more sophisticated ways, enabling the start of the mourning process.

### The Need for Retreat

For several of the participants in this study, having suddenly found themselves in a state of dependency and helplessness stirred up deep feelings of embarrassment and anger. As the perspective shifts from total loss to partial loss, participants saw their amputation as a horrible defect that is to be pitied and looked down upon. This state of humiliation rendered the much-needed post-surgery support of family and friends into a pitiful situation, difficult to tolerate for the wounded self. It is perhaps one reason why participants felt the need to isolate themselves, whenever possible, from human contact. Being upset when stared at by others, perceiving one’s body as defective and the need for isolation are common findings among studies in this field (Ostler et al., 2014; Khan et al., 2018). Their need to avoid contact with other people seemed to serve important temporary defensive functions for the vulnerable recovering self, shielding it from experiencing shame and hostility as a result of anticipation of negative attitudes or actual negative attitudes of others (Murray and Forshaw, 2013). This finding is supported by other authors (Bergo and Prebianchi, 2018) who consider that separation from social networks (a recurring theme among amputees) is temporary, being specific to the immediate post-operative period. With time and especially after prosthesis, the patient becomes accustomed to the situation and begins to resume the old connections.

### Living in One’s Own Head

However, necessary isolation may be in the beginning, it also has the potential to become a pathological element. Lack of significant human interaction means lack of a healthy feed-back loop, of opportunities for changes in one’s perspective. Thus, the mind is left to answer its own questions, to circle its own fears and unknowns. The scope of existence narrows and several participants in our study were struggling not to “fall prey to despair.” For two elderly participants this struggle seemed lost, as they could find no rewarding perspectives of their future. Ruminations about their own guilt in bringing about the amputation was another factor that caused emotional distress. Anxiety was also present in some participants, mainly regarding the uncertainty of the future, being alone, losing their jobs and means of existence. This uncertainty was manifested as a sense of threat, of imminent danger that compels vigilance and thus caused insomnia, negative cognitions, rumination, stress, and irritability.

Almost all previous studies on the psychological aspects of amputation reported a noticeable decrease in the psychological well-being and quality of life (Desmond and MacLachlan, 2002; Schofield et al., 2006; Waqar et al., 2015; Ali et al., 2017; Khan et al., 2018). According to some authors depression and anxiety are moderately high up to 2 years after amputation (Waqar et al., 2015) and so the risk of depression does not diminish as an amputation becomes more remote in time (Rybarczyk et al., 2004). From our theoretical viewpoint, the existence of an adequate interpersonal relationships system (family and friends) is critical in providing the self-object needs that the development of a healthy self requires, including the need for security and soothing. Here, we consider that a basic training of family members regarding medical and psychological issues associated with amputation is recommended and can encourage more realistic and balanced attitudes and responses, while diminishing the risk of actually amplifying existing feelings of helplessness, anger, and despair.

### A New Frustrating Reality

In our study anger was often the response to realizations of constraints and limitations that the new condition brought to the participants. In some cases, the perceived unfairness of the trauma exacerbated feelings of anger and opened the way for resentment and envy towards other people. For others, confronting their impaired capacity to fully engage in previous daily activities and roles was a great frustration that left them feeling angry and bitter. Also, the realization that previous pleasurable activities and hobbies were now off limits was a source of great distress. Another hindering aspect for the participants in our study was the difficulty associated with discovering and integrating the limits of the new body scheme within the self, in terms of functionality. Phantom limb sensations coupled with automatic reactions were often the cause of cognitive dissonance and even injury, as was the case with one of the participants. Pain from the phantom limb may also exacerbate feelings of depression (Murray and Forshaw, 2013), cause mood dysregulation (Trevelyan et al., 2016) and predispose the person to accidents (Senra et al., 2012). Generally, these false sensations are reported in 50–85% of amputees, with varying intensity and duration, spanning weeks or months after surgery (Margalit et al., 2013). This aspect of amputation generates frustration and stress and it can become a threat to the rehabilitation process and the psychological and physical well-being of the person.

### (Not So) New Horizons

Despite obstacles, some the participants in our study showed an increased desire to return to previous roles and activities. This seemed to provide a shielding effect from the negative effects of the trauma. The need for self-restoration, as manifested through resuming previous activities, can be seen as a denial of reality but at the same time it provided participants with the opportunity to adjust and also re-instantiate connections with their former ambitions and goals. This helped them somewhat establish a sense of continuity. Adjustment and regaining a sense of normality is often seen as a re-negotiation of self-identity (Hamill et al., 2010; Senra et al., 2012). This means aligning the “internal self,” the existing self-representation, to the “external self,” the person with a disability and altered body image. This process could be more effective if there is an opportunity to resume previous roles, given that the additional constraints are not entirely prohibitive. In such circumstances, the new reality might be less alienated, less disconnected from past experience thus facilitating psychological health and enabling the person to access available internal and external resources. These findings are supported by previous results (Murray, 2010; Dunne et al., 2014; Zhu et al., 2020) showing that the desire to connect with previous interests and activities was a way to recover a feeling of normality. From our theoretical viewpoint, involving the amputee in a process of reality negotiation (Carpenter, 1994) and the gradual accommodation of a new self-representation is crucial. In time, the integration of trauma is possible thus further strengthening the continuity in the sense of self by decreasing dissociation. For example, one participant felt that his efforts toward resuming previous activities were being sabotaged by his family which gave him serious distress. Hence, these findings suggest that such endeavors on the part of amputees should be encouraged and provided with an adequate support and oversight.

### In or Out

In our study, the degree to which one has sufficiently internalized self-object functions or relies on external sources of sustainment, seems to be linked to the mourning process, consequent adaptation capacity, and psychological well-being. Acknowledging the trauma and focusing on current resources and faith in one’s strengths seemed to have a positive effect for some participants. Those that were more optimistic about the future were more inclined to explore existing possibilities for improving their condition and to engage their environment. For example, one participant had modifications done to his vehicle in order to be able to drive, while another found hope in the idea of having prosthesis and resuming physical activities in the future. Previous studies (Hall et al., 2005; Zhu et al., 2020) have had similar findings regarding the rehabilitation process. They found that the ability to self-manage and make adjustments influenced how easily one could regain a sense of normality and balance in their lives.

The support of family, friends, and colleagues, as well as faith in God were pillars of strength for one participant. In her situation, the source of self-cohesion came from outside, from people who provided essential functions of validation and appreciation, and through them, the belief in the divine was maintained. For another participant, the strength to overcome obstacles came from her fear of traumatizing her supportive daughter. For her, succumbing to adversity was not an option. Similar to findings in other studies (Murray and Forshaw, 2013; Khan et al., 2018), some participants in our research benefited from the assistance of friends and family, especially in the restoration of the sense of self-worth. For another participant, the interventions of the family were felt as overprotective, this being in line with the idea that the quality of the relationships is of significant importance (Hamill et al., 2010). The feeling of abandonment by friends and family was also present among our participants. This comes to reinforce the fears of participants of being left alone to dread the existential uncertainty. A complex picture about the experience of participants emerges here, one that takes into account apparently contradictory needs, such as need for isolation, passive-dependent needs and need for autonomy. From a Self psychology perspective, this becomes less paradoxical if all these needs are allowed to manifest in the context of a supporting self-object matrix.

Assessing the dynamics of the self, in relation to self-object needs, could prove very useful in determining the kind of support one requires in the case of amputation. These findings could encourage specialists working with amputees to take into consideration case by case evaluations form a Self psychology perspective in order to develop targeted plans for individual recovery.

Regardless of the circumstances that led to amputation, it brings a dramatic change in the life of the individual that undergoes a shock phase, acceptance and finally adapting to the new situation. But long after surgery, patients, in addition to physical and mental suffering, face a number of difficult problems: the cost of the prosthetic limb, the pursuit of compensation in the case of car or work-related accidents, continuing treatment for chronic disease (such as diabetes), decreased sexual activity, especially in young people, and, in particular, uncertainty about the future. The latter is the most commonly reported feeling (Galván et al., 2009; Sales et al., 2012; Simsek et al., 2017; Bergo and Prebianchi, 2018) and it was also present in the findings of our study. It stands to reason that in order for the person to regain a sense of normality and to be able to develop an adaptive new self-representation as a participative active being in the environment, a complex system of support and resources must be in place.

## Conclusion and Limitations

This study presents an idiographic analysis, wishing to draw attention on the need to develop intervention and support plans by physicians and therapists for patients who have undergone amputations, based on identifying the psychological consequences of limb amputation.

In a qualitative study (Franchini and Savoia, 2013; Bergo and Prebianchi, 2018), it was observed that patients who participated in group psychotherapy became more independent and showed greater acceptance, faster rehabilitation, and positive thinking. Therefore, the efficacy of such programs may be enhanced if the practitioners know the phenomenology of the various psychological consequences in the case of patients with limb amputation. Presenting to patients the situations of people who have successfully adapted to this disability and direct interaction with such people, often result in providing inspirational motivation, optimism, and balance.

Also, the present study wants to raise awareness among medical staff about the psychological problems faced by amputees. Understanding them, the trauma associated with amputation, the loss and the permanent suffering will generate attachment, respect and compassion, improving the medical approach. Consequently, it is necessary to set up a multidisciplinary team (surgeons, neurologists, psychologists, physiotherapists, and orthotists) in order to develop informed interventions aimed at diminishing the undesirable effects of amputation and enabling the patient to resume a normal life. In this respect, a Self psychology perspective could help to better differentiate the psychological needs that individuals have in regard to their adjustment process. Also, this approach could also be useful in informing family members, caregivers, or healthcare professionals about how empathic understanding of the amputee’s experience can facilitate appropriate responses, this in turn providing a more adequate environment for the gradual restoration of self-agency and self-worth, while diminishing the risk of negative reactions.

A closely related approach to the one proposed here is founded in the Self-determination theory (SDT) developed by Deci and Ryan (1985). This macro theory of human motivation and personality has focused on need support as a process that facilitates internalization (autonomous self-regulation) and the development of adequate motivation and well-being. Over the last two decades, research testing the applicability of SDT within health contexts has provided good evidence for a variety of health outcomes including depression, anxiety, somatization, and quality of life (Williams and Deci, 2001; Ryan et al., 2008; Williams et al., 2009; Ng et al., 2012; Chemtob et al., 2019). One of the main features of SDT is its understanding of the importance that the social context plays in the process of internalization. Self-psychology, as a developmental model, goes one step further and provides a higher resolution framework that can help practitioners in health care contexts better connect to the patient-professional experience. We think this aspect is useful since, given the time constraints one usually encounters in the medical system, designed interventions, focused on types of action and language, for example, may acquire a stereotypical quality.

However, the limitations of this study warrant further investigation. First, the small number of participants limits the ability of this study to touch upon different types of experiences regarding amputation and possible differences that could emerge due to age, gender, socioeconomic and ethnic background, cause and type of amputation or time elapsed since amputation. However, the aim of IPA is to investigate how individuals experience a particular phenomenon and, as such, a sample as the one used in our study is justified. Second, the relative short amount of time passed since amputation narrows the scope of the research and leaves out opportunities for exploring subsequent themes. A follow-up study would be needed in order to assess the outcomes of the participant’s adjustment efforts. Nevertheless, this time frame provided the opportunity to extract a more actual and accurate account of the experience and psychological impact of amputation.

All things considered, the findings in this study support the existing literature and also offer insight into how self-representation is affected by the loss of a limb. Also, it draws attention to the complexity of the adjustment process and the need for addressing it on multiple levels of analysis.

## Data Availability Statement

The datasets presented in this article are not readily available because of the sensitive and confidential nature of the information. Requests to access the datasets should be directed to catalina.rosca@politice.ro.

## Ethics Statement

The study was conducted according to the guidelines of the Declaration of Helsinki, and approved by the Institutional Ethics Committee of NUPSPA (protocol code no. 145, April 01, 2019). All respondents were made aware of the procedures and gave verbal consent to participation. Written informed consent from the participants was not required to participate in this study in accordance with the institutional requirements.

## Author Contributions

All authors have made substantial and equal contribution in all stages of the present study. All authors read and approved the final version of the paper.

### Conflict of Interest

The authors declare that the research was conducted in the absence of any commercial or financial relationships that could be construed as a potential conflict of interest.
